# Development of Carbofuran Imprinted Electrochemical Sensor Based on Carbon Black–Graphitic Carbon Nitride Nanocomposite and Carbofuran Detection in Orange Juice Samples

**DOI:** 10.3390/foods15183313

**Published:** 2026-09-19

**Authors:** Didem Gün, Dilek Doğan, İlknur Polat, İsmail Hakkı Akbudak, Mehmet Lütfi Yola

**Affiliations:** 1 Department of Basic Sciences, Faculty of Engineering, Hasan Kalyoncu University, Gaziantep 27010, Türkiye; didem.gun@hku.edu.tr; 2 Department of Nutrition and Dietetics, Faculty of Health Sciences, Hasan Kalyoncu University, Gaziantep 27010, Türkiyeilknur.polat@hku.edu.tr (İ.P.); 3 Division of Intensive Care Medicine, Department of Internal Medicine, Faculty of Medicine, Pamukkale University, Denizli 20160, Türkiye; iakbudak@pau.edu.tr; 4 Department of Biology, Faculty of Science, Ankara University, Ankara 06100, Türkiye; 5 Integrated Technologies Research Center (BUTAM), Ankara University, Ankara 06690, Türkiye

**Keywords:** carbofuran, carbon black, graphitic carbon nitride, molecularly imprinted polymers, polypyrrole, electrochemical sensor, food analysis

## Abstract

Carbofuran (CAR) is an extremely toxic carbamate insecticide that poses serious threats to both environmental and human health, which necessitates highly sensitive analytical methods for its detection in food matrices. This research introduced a novel strategy for CAR detection, presenting the synergistic interaction between molecularly imprinted polymers (MIPs) and a carbon black–graphitic carbon nitride (CB-g-C_3_N_4_) nanocomposite. By integrating the high-affinity, specific recognition capabilities of MIPs with the superior electrochemical sensing performance of the CB-g-C_3_N_4_ platform, the developed methodology significantly improved the sensitivity and selectivity in pesticide analysis. Following the modification of the glassy carbon electrode (GCE) with the CB-g-C_3_N_4_ nanocomposite, the CAR-imprinted sensing interface was fabricated via cyclic voltammetry (CV). The electropolymerization process was conducted in a solution containing 100.0 mM pyrrole (Py) as the functional monomer and 25.0 mM CAR as the template molecule. This controlled electrochemical deposition facilitated the formation of a selective MIP layer, which effectively provided specific recognition sites on the nanocomposite-modified surface. Under optimized conditions, the sensor exhibited a linear dynamic range ranging from 1.0 × 10^−9^ M to 5.0 × 10^−8^ M. Furthermore, the limit of detection (LOD) was calculated to be 3.0 × 10^−10^ M, which highlights the sensor’s high sensitivity and its suitability for monitoring pesticide traces. To evaluate the practical utility of the proposed electrochemical sensor, its performance was investigated using orange juice samples. The analytical results demonstrated excellent recovery rates close to 100%, confirming the sensor’s ability to operate effectively within complex food matrices.

## 1. Introduction

Carbamate-based pesticides are widely used in agricultural pest management, providing essential protection for major commodity crops such as cotton, maize, wheat, rice, and various vegetables. Within this chemical class, CAR (2,2-dimethyl-2,3-dihydro-7-benzofuranyl N-methylcarbamate) has emerged as a predominant choice for agricultural applications owing to its high insecticidal potency [1]. Despite its efficacy, CAR exhibits high water solubility (0.70 g L^−1^), significant resistance to degradation, and high mobility within soil and aquatic environments, which facilitates its entry into and subsequent accumulation within food chains. This environmental persistence triggers substantial metabolic transformation, during which approximately 80.0% of the parent compound is rapidly metabolized into 3-hydroxycarbofuran, while more than 5.0% is subjected to conversion into 3-oxycarbofuran [2]. Even at trace concentrations, CAR and its toxic metabolites can inhibit cholinesterase activity, impair endocrine and neurological functions, and thereby pose significant risks to human health, wildlife populations, and overall ecosystem integrity [3]. CAR has been prohibited in numerous countries; nevertheless, it continues to be used in agricultural practices. In response to concerns regarding human and ecological safety, the regulatory authorities have set maximum permissible levels for CAR residues in both water and food. The World Health Organization, for example, has proposed a guideline value of 0.007 mg L^−1^ for CAR. These regulations underscore the critical need for highly sensitive and reliable analytical methods capable of detecting CAR in complex environmental matrices, including food products. Conventional analytical methods for CAR quantification—including high-performance liquid chromatography, spectrophotometry, and fluorescence assays—offer high precision. However, they possess significant disadvantages such as high costs, difficult sample preparation protocols, and an inability to facilitate rapid on-site detection. Consequently, electrochemical sensors have gained considerable attention as an alternative method, offering a cost-effective, sensitive, and user-friendly approach suitable for real-time monitoring applications [4,5,6].

Owing to their distinctive physicochemical characteristics, the nanocomposites have attracted significant attention for electrochemical analysis because these materials facilitate the electron transfer kinetics while simultaneously increasing the number of electroactive sites at the electrode–solution interface. Moreover, the synergistic integration of these materials within various sensing platforms enhances the overall electrocatalytic performance of the system. By acting as electrode modifiers in electrochemical sensing applications, nanomaterials significantly improve both sensitivity and selectivity, and their superior catalytic and adsorptive capabilities can facilitate precise molecular recognition [7]. CB has emerged as a significant carbon-based material with a wide range of utilities in electroanalytical applications due to its exceptional electrical conductivity and rapid electron transfer kinetics, which are facilitated by its extensive surface area, chemical robustness, and inherent structural defects. Furthermore, the material’s intrinsic properties make it an excellent candidate for functionalization or strategic modification with a diverse range of chemical compounds and auxiliary materials [8]. Furthermore, the utilization of CB in the construction of three-dimensional electrodes offers several advantages, such as the high stability, catalytic efficiency, and analytical reproducibility of the resulting sensor systems [9]. g-C_3_N_4_ is characterized as a two-dimensional, polymeric, carbon-based material. Its structural framework consists of carbon and nitrogen atoms linked via sp^2^ hybridization within tri-s-triazine units. Furthermore, the presence of conjugated π-π interactions between these rings facilitates semiconducting behavior. The polymeric framework of g-C_3_N_4_ is distinguished by a high specific surface area, robust chemical stability, and notable energy storage capabilities. Furthermore, the presence of lone-pair electrons associated with the nitrogen atoms within its structure results in significant catalytic activity of the material [10]. Beyond its intrinsic properties, g-C_3_N_4_ has been successfully integrated into the architecture of three-dimensional (3D) electrodes. Within these configurations, the g-C_3_N_4_-based network serves to enhance the catalytic performance across a variety of electrochemical platforms [11].

Molecular imprinting is a highly effective strategy for synthesizing specialized polymers through a sequential three-stage process. To initiate the process, functional monomers interact with a target template molecule, forming a stable complex through either covalent or non-covalent interactions. The procedure continues with the polymerization of this complex, followed by the final stage where the template is extracted by employing a suitable solvent. This process creates a polymeric framework with cavities designed for the selective recognition of the original analyte. Thus, MIPs are widely used for their exceptional selectivity, combined with the practical advantages of cost-effectiveness, high durability, and relative ease of fabrication [12,13,14].

This study presents an innovative and highly efficient methodology for the precise quantification of CAR pesticide. The core of this approach involves the synthesis of a CB-g-C_3_N_4_ nanocomposite. This material is integrated into a GCE, which is subsequently functionalized with MIPs. The GCE is selected as the working electrode due to its advantageous characteristics, including a wide potential window, chemical inertness, and surface renewability. The performance of this modified electrochemical sensor is successfully validated through the accurate quantification of CAR in orange juice samples, providing a valuable contribution to the development of advanced sensing technologies.

## 2. Materials and Methods

### 2.1. Materials and Instrumentation

CAR, dopamine (DOP), ascorbic acid (AA), fenitrothion (FEN), melamine (MEL), ethylcellulose (ETH), terpineol (TER), carbon black (CB), Py monomer, phosphate buffer, and sodium chloride (NaCl) were procured from Sigma-Aldrich (St. Louis, MO, USA). Scanning electron microscopy (SEM, ZEISS EVO 50 SEM, Tokyo, Japan), Fourier Rigaku X-ray diffractometer (XRD, Tokyo, Japan), and DeltaNu Examiner Raman microscope (Tokyo, Japan) with a 785 nm laser source were used for structural characterization. Isotech Annealing Furnace (from 30 to 1000 °C), model 414 (Merseyside, UK), was used for g-C_3_N_4_ synthesis. The measurements of electrochemical impedance spectroscopy (EIS), square wave voltammetry (SWV), and cyclic voltammetry (CV) were performed using a GAMRY Reference 600 workstation equipped with a C3 cell stand (Wuhan, China). The frequency of 15 Hz, amplitude of 25 mV, and potential increment of 4 mV were used for SWV measurements.

### 2.2. Preparation of g-C_3_N_4_ and CB-g-C_3_N_4_ Nanocomposite

The synthesis of the g-C_3_N_4_ nanomaterial was performed in accordance with the established protocols by subjecting 15 g of MEL to thermal treatment at 600 °C within an annealing furnace at a heating rate of 5 °C min^−1^. After a duration of 5 h, a greenish-yellow product (g-C_3_N_4_) was subsequently ground into a fine powder using a mortar and pestle [15,16].

To prepare the CB-g-C_3_N_4_ nanocomposite, 4 g of ETH was first dissolved into 25 g of TER and stirred continuously at 90 °C for approximately 3 h until the binding solution was homogenized. After that, the nanocomposite was prepared in the mole ratio of 50%g-C_3_N_4_ and 50%CB in the presence of the binding solution (10.0 mL). Lastly, a homogeneous mixture was achieved through grinding and mechanical dispersion for 1 min. Moreover, the selection of a mole ratio of 50%g-C_3_N_4_ and 50%CB resulted from the necessity to balance electrical conductivity with high surface area and specific active site availability. CB served as a robust conductive scaffold that facilitated rapid electron transfer and reduced the charge-transfer resistance of the electrode surface, while g-C_3_N_4_ provided a nitrogen-rich, two-dimensional structure that enhanced the adsorption capacity for CAR and stabilized the imprinted polymer matrix. This balance was disrupted in other mole ratio selections.

### 2.3. Development of CB-g-C_3_N_4_ Nanocomposite Modified GCE (CB-g-C_3_N_4_/GCE)

The GCE with a geometric area of 0.027 cm^2^ was initially cleaned according to the literature protocols [17]. GCE was subjected to a rigorous cleaning and polishing protocol to ensure a mirror-like surface finish. This was achieved by initially abrading the electrode surface with fine-grain wet emery paper (4000 grit size), followed by sequential polishing using 0.1 µm and 0.05 µm alumina slurries (supplied by Baikowski Int. Corp., Charlotte, NC, USA) applied on microcloth pads (procured from Buehler, Lake Bluff, IL, USA) to maintain consistent surface morphology and high electrochemical performance. To ensure optimal surface cleanliness and remove any residual contaminants, the GCE underwent a multi-stage sonication procedure. Initially, the electrodes were sonicated twice in ultrapure water, followed by immersion in a 50:50 (*v*/*v*) mixture of isopropyl alcohol and acetonitrile. To eliminate trace alumina particles, the surfaces were thoroughly rinsed with water and subjected to brief ultrasonic cleaning (Bandelin RK 100, Berlin, Germany). Finally, the GCE was rinsed with pure acetonitrile to ensure the complete removal of any physisorbed or unreacted materials, thereby preparing a pristine surface for electrochemical application. Before modification, the electrodes were dried with an argon gas stream for 30 min. The electrode surface was then modified by depositing 20.00 μL of a 2.00 mg mL^−1^ CB-g-C_3_N_4_ nanocomposite aqueous dispersion, followed by solvent evaporation using an infrared lamp to yield the CB-g-C_3_N_4_/GCE. For comparative analysis, a control electrode (CB/GCE) was fabricated using an identical drop-casting procedure with a pure CB aqueous dispersion of the same volume and concentration.

### 2.4. Preparation of CAR Imprinted Sensor and CAR Removal

To fabricate the CAR-imprinted CB-g-C_3_N_4_/GCE, CV was performed for 20 cycles within a potential range of +0.0 V to +1.00 V inside an electrochemical cell containing 100.0 mM Py monomer and 25.0 mM CAR at 25 °C. Py was selected due to its advantageous electrochemical characteristics, such as high conductivity, effective electropolymerization, and enhanced physical stability [18]. During the electropolymerization process, the electrochemical peak signals observed near +0.70 V in the first scan progressively diminished with increasing cycles. The attainment of stable electrochemical signals at a minimum current level indicated the successful formation of the polymer layer on the electrode surface, yielding the MIP/CB-g-C_3_N_4_/GCE. A control NIP/CB-g-C_3_N_4_/GCE was prepared using an identical methodology but omitting the CAR molecule at 25 °C. All electrochemical measurements were conducted using a saturated Ag/AgCl/KCl reference electrode and a Pt wire as the counter electrode. The prepared electrodes (MIP/CB-g-C_3_N_4_/GCE and NIP/CB-g-C_3_N_4_/GCE) in this study were kept in a sealed box to protect them from pressure and temperature changes.

To extract the CAR molecules from the imprinted polymer matrix, a 1.0 M NaCl solution was employed as the desorption medium to effectively disrupt the electrostatic forces and hydrogen bonds between the monomer and the analyte. The MIP/CB-g-C_3_N_4_/GCE was submerged in 10.0 mL of 1.0 M NaCl elution medium for 20 min, and the resulting imprinted electrode was thoroughly dried under vacuum conditions at 25 °C.

### 2.5. Sample Preparation

The orange juice sample, including sugars, acids, polyphenols, etc., was purchased from the supermarket in Türkiye. To prepare the orange juice sample for analysis, 15.0 mL of the juice was first transferred to a 50.0 mL conical flask and centrifuged for 10 min at 5000 rpm. Subsequently, the obtained clear supernatant was diluted with a phosphate buffer (pH 7.0) (1:100) to ensure the CAR analyte concentration was within the linear range of the electrochemical system for SWV measurements at 25 °C. The prepared samples were stored in a sealed box at 25 °C. SWV measurements were performed on 6 independent samples to ensure high accuracy and precision, and results were given as Mean ± Standard error (Section 3.6).

## 3. Results and Discussion

### 3.1. Characterization of CB-g-C_3_N_4_ Nanocomposite

**Figure 1:**
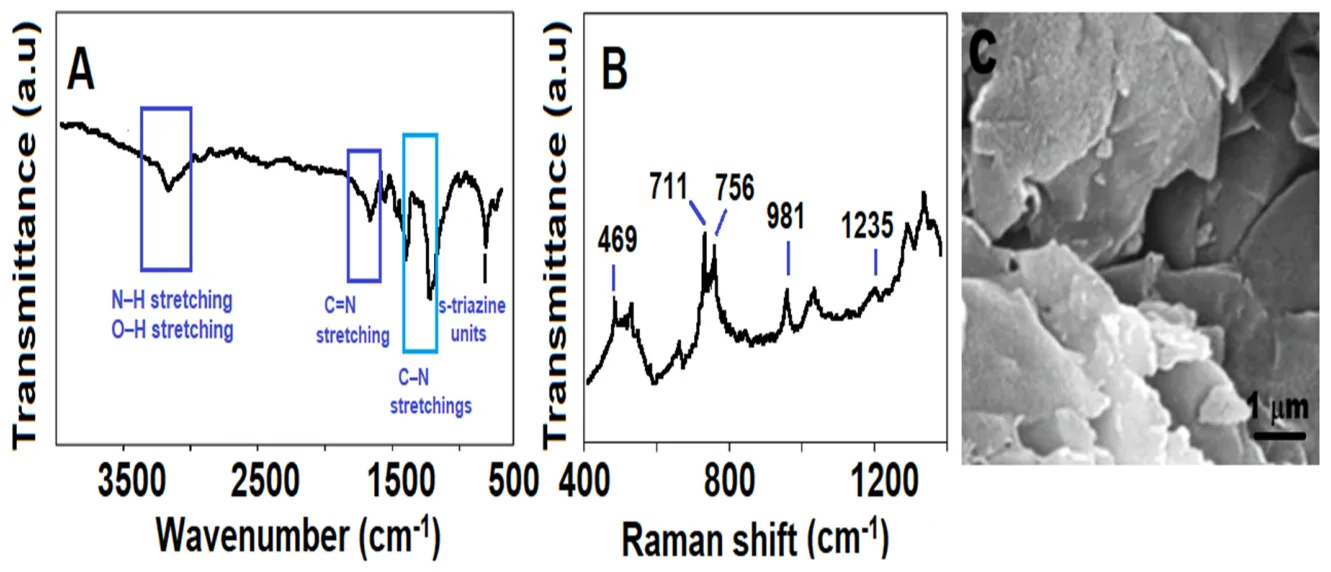
(**A**) FTIR, (**B**) Raman spectral patterns, and (**C**) SEM image of the g-C_3_N_4_ nanomaterial.

The chemical structure of g-C_3_N_4_ was verified through FTIR and Raman spectroscopic analyses. As shown in Figure 1A, the FTIR spectrum exhibited the typical features of g-C_3_N_4_, including a pronounced band at 811 cm^−1^ corresponding to s-triazine ring units in its framework [19]. Furthermore, the absorption peaks identified at 1240, 1329, 1410, and 1459 cm^−1^ were assigned to the characteristic stretching vibrations of C–N bonds present in the g-C_3_N_4_ framework. The vibrational signals identified at 1549 and 1629 cm^−1^ were characteristic of C=N stretching, while the wide spectral region between 3000 and 3400 cm^−1^ indicated the incorporation of N–H and O–H stretching modes in the material structure [15]. Figure 1B presents the Raman spectrum of g-C_3_N_4_. The band at 1235 cm^−1^ was associated with melon polymerization, indicating the condensation of melon-derived units into the g-C_3_N_4_ framework at 600 °C. In addition, a signal at 981 cm^−1^ verified the presence of s-triazine moieties in the structure, whereas the peaks located at 756, 711, and 469 cm^−1^ were characteristic features attributable to the g-C_3_N_4_ lattice [20].

The SEM image of g-C_3_N_4_ clearly revealed its characteristic morphology. As depicted in Figure 1C, the nanomaterial displayed flat and wavy surfaces without agglomeration, indicating the presence of the stacked nanolayers or nanosheets [15,21]. Figure S1 presents the SEM images of the CB and CB-g-C_3_N_4_ nanocomposite, demonstrating a uniform dispersion of CB within the g-C_3_N_4_ matrix and the preservation of their original morphology without any discernible structural alteration [15]. Table S1 presents the EDX analysis results, detailing the compositions of the carbon and nitrogen elements. Pure g-C_3_N_4_ showed a carbon-to-nitrogen ratio of 0.85, suggesting successful synthesis of g-C_3_N_4_. Incorporating CB into pure g-C_3_N_4_ resulted in a substantial increase in carbon content. Conversely, the nitrogen content exhibited a decrease as the proportion of the carbon additive was increased. This observation was in good agreement with the SEM results, which suggested that higher carbon concentrations facilitated a more extensive dispersion of g-C_3_N_4_ across the carbon substrate.

### 3.2. Electrochemical Characterizations of CB and CB-g-C_3_N_4_ Nanocomposite Modified Electrodes

**Figure 2:**
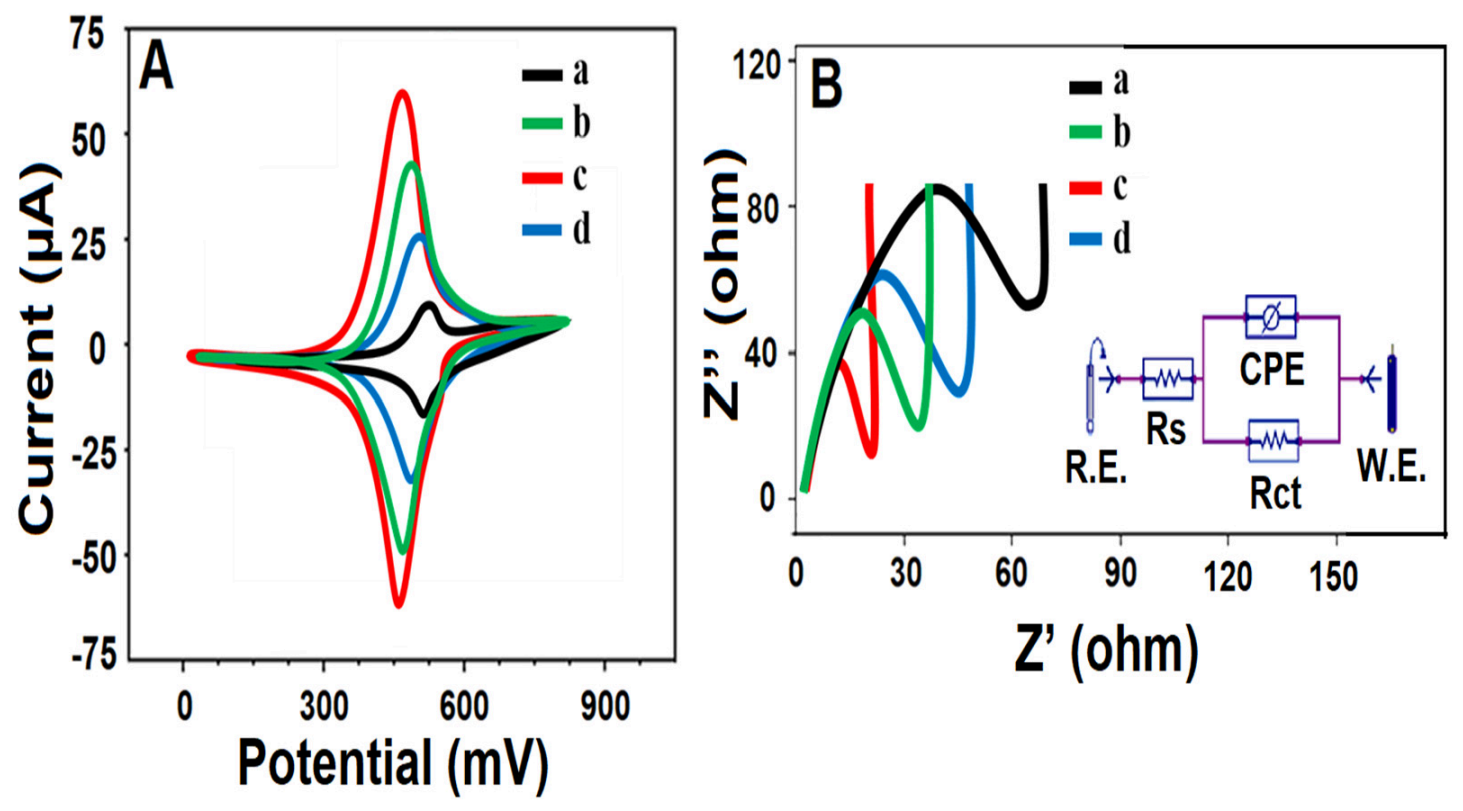
(**A**) CV curves and (**B**) EIS responses at (a) bare GCE, (b) CB/GCE, (c) CB-g-C_3_N_4_/GCE, and (d) MIP/CB-g-C_3_N_4_/GCE upon the successful desorption of CAR molecules (CV was conducted using 1.0 mM [Fe(CN)_6_]^3−/4−^ in a 0.1 M KCl solution as the redox probe at a scan rate of 100 mV s^−1^, while EIS measurements were performed across a frequency interval ranging from 100,000 Hz to 0.1 Hz, utilizing a wave amplitude of 10 mV at a fixed formal potential of 0.160 V). RE and WE denote the reference and working electrodes, respectively.

The electrochemical behavior of the electrodes modified with CB and the CB-g-C_3_N_4_ nanocomposite was evaluated through CV (Figure 2A) and EIS (Figure 2B) analyses. It was observed that the CB/GCE (curve b) exhibited more intense anodic and cathodic peaks compared to the bare GCE (curve a), which served as evidence of significantly enhanced catalytic activity. This superior electrochemical performance was ascribed to the high electrical conductivity, rapid electron transfer, and chemical stability of the CB [8]. Furthermore, the CB-g-C_3_N_4_/GCE (curve c) displayed more intense electrochemical peaks, resulting from a synergistic interaction between CB and g-C_3_N_4_ that facilitated a marked enhancement in charge transfer efficiency [22]. Upon the removal of the CAR template molecules, the MIP/CB-g-C_3_N_4_/GCE (curve d) displayed well-defined anodic and cathodic peaks in the presence of a 1.0 mM [Fe(CN)_6_]^3−/4−^ redox probe. However, the notably lower peak intensities observed for this electrode compared to those of the CB/GCE and CB-g-C_3_N_4_/GCE confirmed the effective deposition of the selective polymeric layer. This film functions as a barrier that impedes electron transfer between the solution-phase probe and the electrode surface, thereby increasing the charge transfer resistance and decreasing the current signal. Furthermore, the physical presence of the polymer matrix and the formation of these imprinted recognition sites effectively hindered the mass transfer of electroactive probes toward the electrode surface.

To validate the CV results, EIS was employed to construct Nyquist plots for the bare GCE, CB/GCE, CB-g-C_3_N_4_/GCE, and the CAR-removed MIP/CB-g-C_3_N_4_/GCE (Figure 2B). For these measurements, six replicates of each electrode were prepared (*n* = 6), yielding charge transfer resistance (Rct) values of 70.0 ± 0.81 Ω, 30.0 ± 0.73 Ω, 20.0 ± 0.90 Ω, and 45 ± 0.43 Ω, respectively. These results highlighted the efficacy of the CB-g-C_3_N_4_ nanocomposite as an electrochemical sensing surface, showing a close alignment between experimental data and the conventional Randles equivalent circuit, which included solution resistance (Rs), Rct, and a constant phase element (CPE) for the GCE and MIP-modified interfaces.

**Figure 3:**
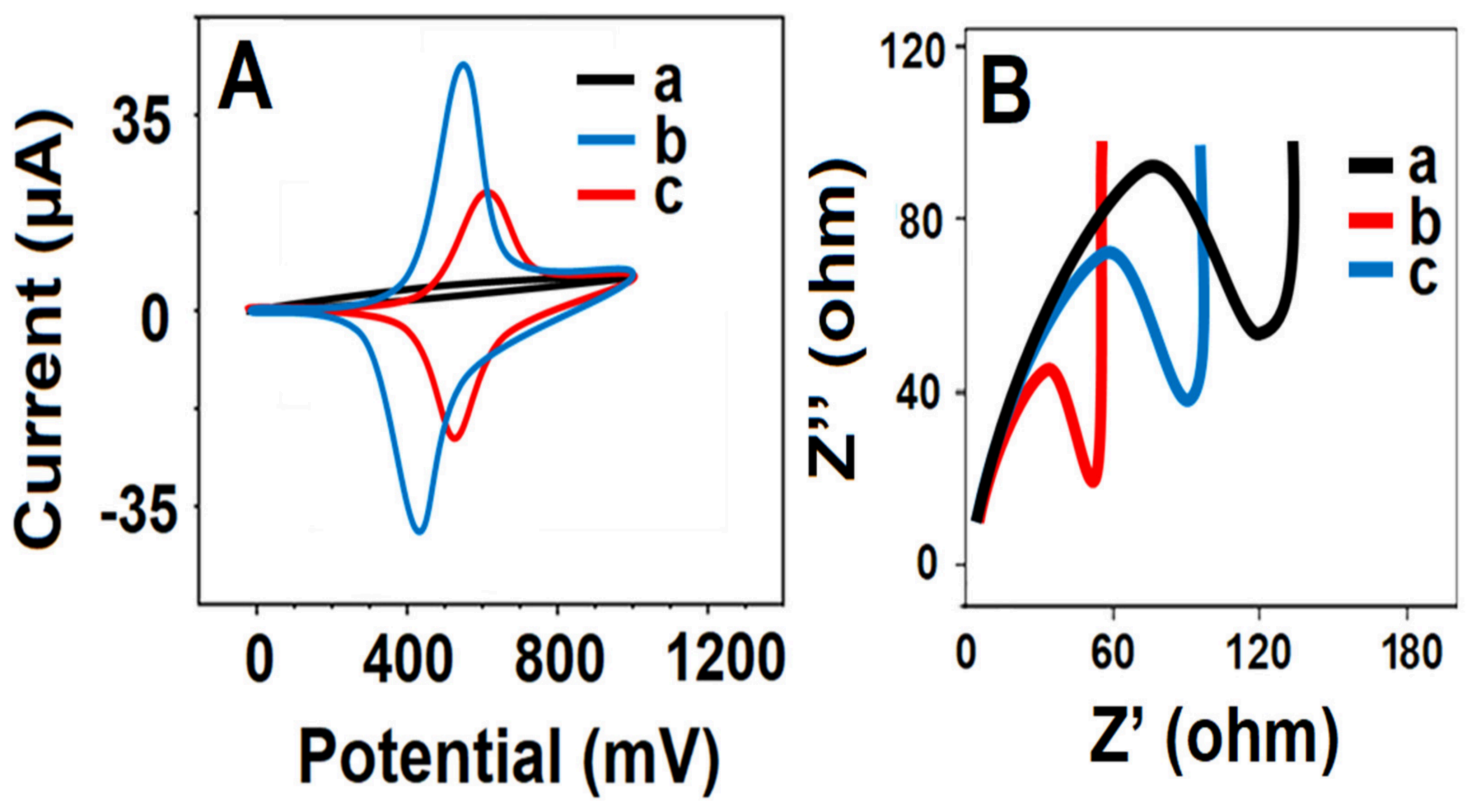
(**A**) CV curves and (**B**) EIS responses at (a) MIP/CB-g-C_3_N_4_/GCE without CAR removal, (b) MIP/CB-g-C_3_N_4_/GCE upon the successful desorption of CAR molecules, and (c) NIP/CB-g-C_3_N_4_/GCE after the rebinding of CAR molecules (CV was conducted using 1.0 mM [Fe(CN)_6_]^3-/4-^ in a 0.1 M KCl solution as the redox probe at a scan rate of 100 mV s^−1^, while EIS measurements were performed across a frequency interval ranging from 100,000 Hz to 0.1 Hz, utilizing a wave amplitude of 10 mV at a fixed formal potential of 0.160 V).

The electrochemical behavior of the MIP and NIP electrodes was systematically evaluated through CV and EIS techniques. As illustrated in Figure 3A, the initial electropolymerization of pyrrole (curve a) led to a significant reduction in both redox peaks [23], while the subsequent removal of the CAR template molecules (curve b) facilitated the recovery of a well-defined peak current, confirming the creation of accessible surface sites. In contrast, the incubation of the NIP/CB-g-C_3_N_4_/GCE in the CAR solution (curve c) resulted in negligible changes in the peak currents.

As shown in Figure 3B, the impedance spectroscopy data were in harmony with the CV results, which were shown in Figure 3A. Notably, the MIP/CB-g-C_3_N_4_/GCE prior to CAR removal demonstrated its highest Rct value (curve a), whereas the same electrode exhibited a reduced Rct value following the desorption of CAR molecules (curve b), which was directly correlated with the enhanced electrochemical activity of the interface. Similarly, the incubation of the NIP/CB-g-C_3_N_4_/GCE in the CAR solution (curve c) demonstrated a slightly lower Rct value compared to curve a. In conclusion, these findings confirmed that the formation of electroactive sites resulting from the desorption of CAR molecules significantly facilitated interfacial electron transfer kinetics.

### 3.3. Development of CAR Imprinted Polymer on CB-g-C_3_N_4_/GCE

**Figure 4:**
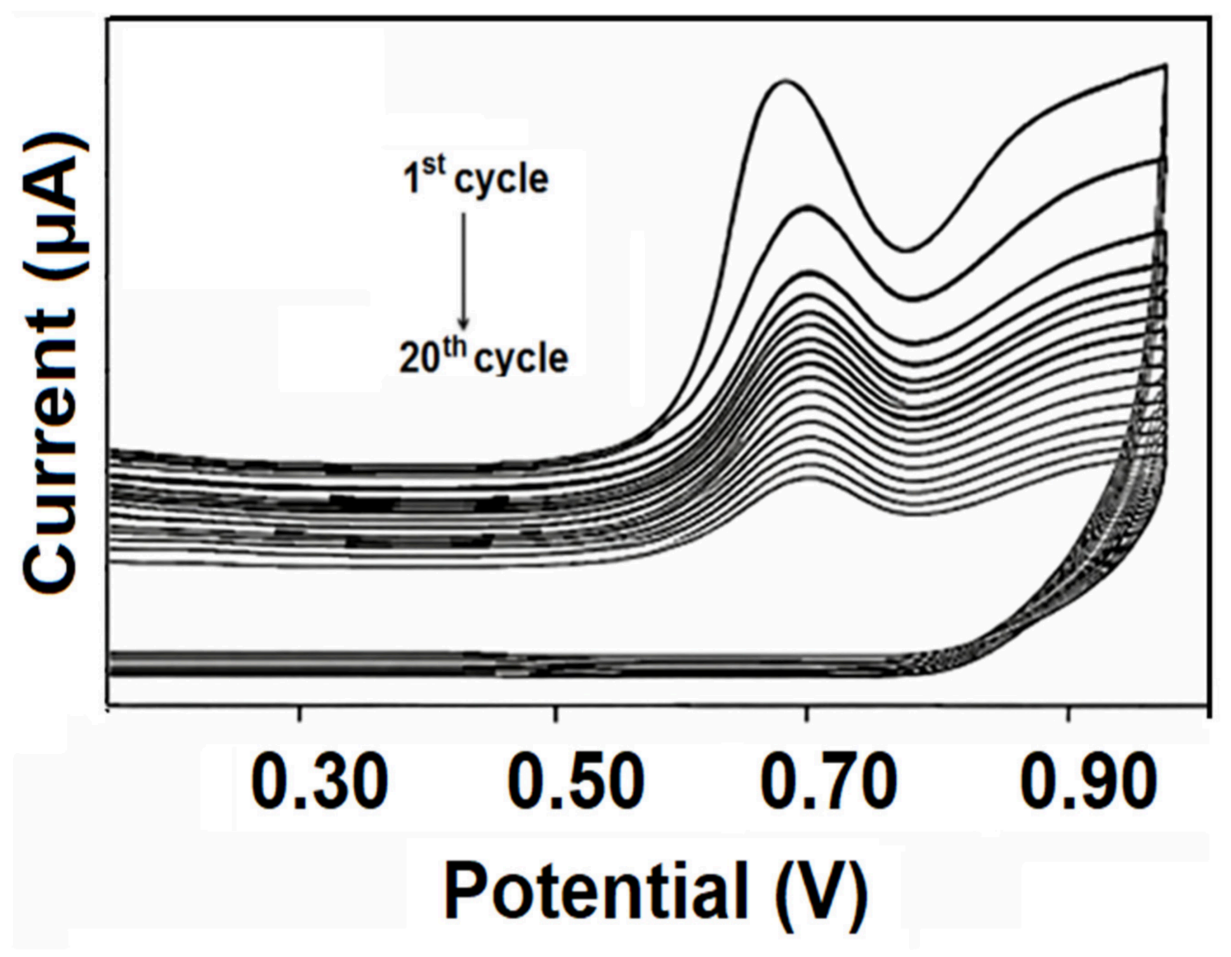
CV polymerization on CB-g-C_3_N_4_/GCE (Potential scan rate: 100 mV s^−1^).

The polymerization process was conducted in an electrochemical cell utilizing a CB-g-C_3_N_4_/GCE working electrode in a mixture of 100.0 mM Py monomer and 25.0 mM CAR, by applying an anodic potential scan from 0 to +1.00 V. According to the data presented in Figure 4, the electrochemical signals during polymerization at +0.70 V exhibited a consistent decline across subsequent scans, ultimately stabilizing by the 20th scan. Furthermore, while the NIP electrode maintained a uniform surface lacking nano-cavities (Figure S2B), the MIP electrode demonstrated a porous structure with distinct accessibility for the targeted recognition of the CAR analyte (Figure S2A).

### 3.4. Optimization

#### 3.4.1. pH Effect

Within electrochemical studies, the electrolyte pH is a fundamental variable that significantly influences the current response. The anodic peak potential exhibited a pronounced shift toward more negative values as pH increased, indicating that protons were directly engaged in the electrochemical oxidation mechanism of CAR, with the regression equation:

(1) Ep (V) = −0.0403 pH + 0.7063 (R^2^ = 0.9993)

The observed slope suggested that the oxidation process at the MIP/CB-g-C_3_N_4_/GCE surface involved an equivalent ratio of electrons and protons. Consequently, the electro-oxidation of CAR was characterized by a proton-coupled electron transfer mechanism, primarily proceeding through the oxidation of the phenolic group via the transfer of one electron and one proton [24]. According to Figure S3A, the peak current increased progressively as the pH was raised from 4.0 to 7.0, reaching its maximum value at a neutral pH level. Beyond pH 7.0, the peak current exhibited a steady decline, likely due to a reduction in proton concentration and alterations in the electrode surface interactions. Therefore, a pH of 7.0 was selected as the optimal medium, as it provided the most favorable equilibrium between electrochemical stability and signal response for the detection of CAR.

#### 3.4.2. Mole Ratio Effect

The sensitivity performance of MIP-based electrochemical sensors is significantly influenced by the thickness of the polymeric layer at the electrode interface. While excessive monomer ratios result in unfavorable non-specific interactions, insufficient ratios diminish the frequency of essential analyte–monomer binding. Consequently, the MIP/CB-g-C_3_N_4_/GCE achieved the maximum peak sensitivity when utilizing an optimal balance of 100.0 mM monomer and 25.0 mM analyte concentrations, as illustrated in Figure S3B.

#### 3.4.3. Scan Cycle Effect

The impact of the number of electropolymerization scan cycles was evaluated within 10 to 50 cycles. The results indicated that exceeding 20 cycles led to the formation of an excessively thick polymeric layer. Therefore, to ensure optimal sensitivity and prevent a signal decrease caused by over-deposition, 20 scan cycles were selected as the optimal protocol for fabricating the MIP-based electrode, as evidenced by the data in Figure S3C.

#### 3.4.4. Elution Time Effect

The duration of the elution process represents a critical determinant of sensor sensitivity in MIP-based electrochemical platforms, as the optimal performance depends on the complete extraction of analyte molecules from the electrode interface. Consequently, the experimental results presented in Figure S3D demonstrated that a 20 min elution period was optimal for the successful removal of template molecules.

### 3.5. Sensitivity of MIP/CB-g-C_3_N_4_/GCE

**Figure 5:**
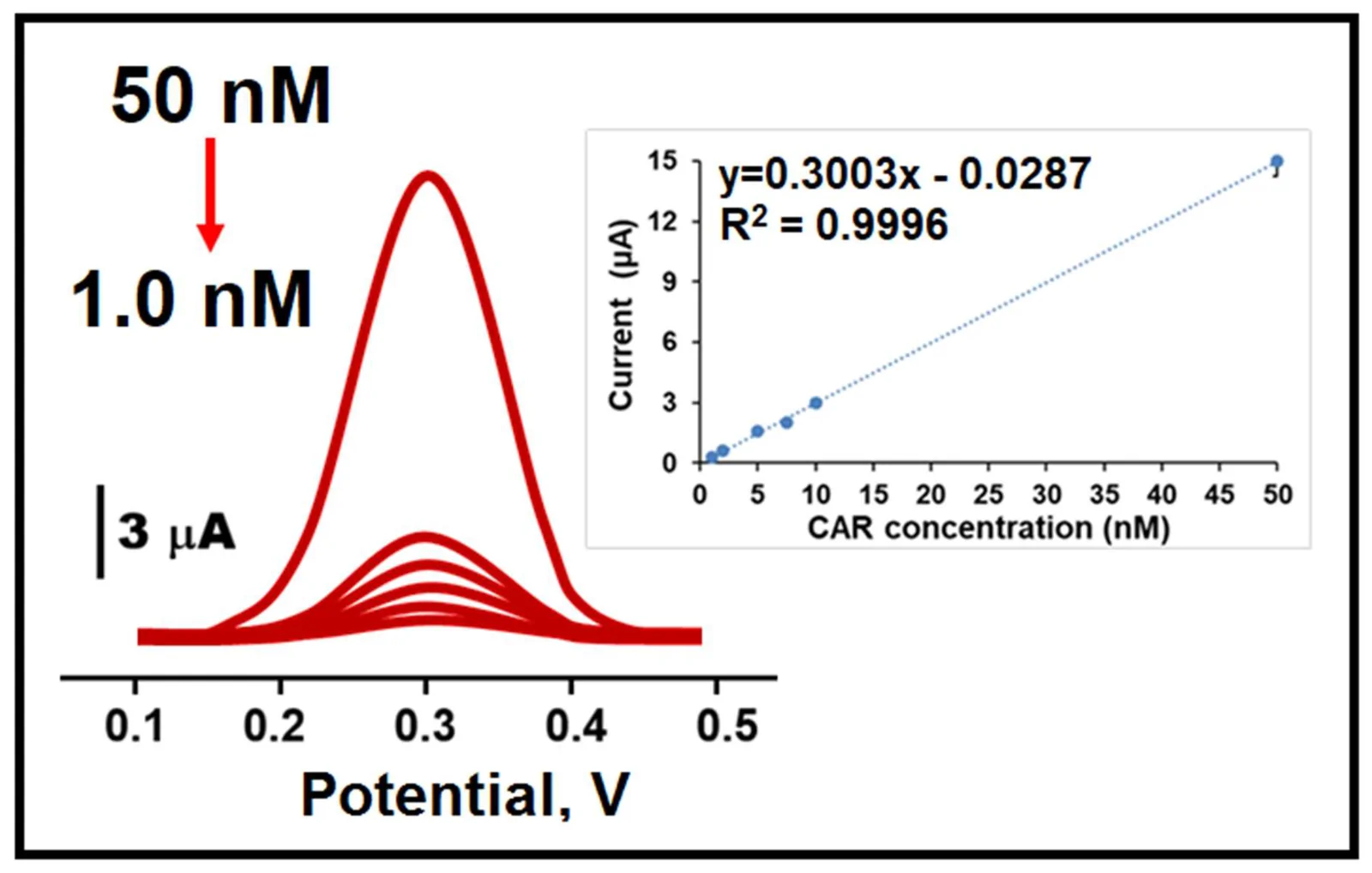
Square wave voltammograms recorded at increasing CAR concentrations (1.0–50.0 nM) using the MIP/CB-g-C_3_N_4_/GCE in phosphate buffer solution at pH 7.0. Inset: Corresponding calibration plot of CAR concentration versus peak current response.

The performance of the MIP/CB-g-C_3_N_4_/GCE electrode was evaluated using SWV, as depicted in Figure 5, revealing remarkable sensitivity toward CAR concentrations ranging from 1.0 to 50.0 nM. A clear linear relationship between the SWV response and analyte concentration was observed, as described by the regression equation y (μA) = 0.3003 × (C_CAR_, nM) − 0.0287 (the inset of Figure 5). This proposed electrochemical sensor exhibited robust analytical performance, achieving a limit of quantification (LOQ) of 1.0 × 10^−9^ M and a LOD of 3.0 × 10^−10^ M (the supporting mathematical expressions were included in the Supplementary Documentation), and the data of the calibration curve for the proposed sensor were given in Table S2. A comprehensive review of the existing scientific literature regarding various analytical techniques for CAR detection was summarized in Table 1. Notably, the MIP/CB-g-C_3_N_4_/GCE sensor demonstrated superior sensitivity compared to previous reports. This enhanced performance was primarily attributed to the synergistic electrochemical properties of the nanocomposite and its superior surface modification capabilities. Specifically, the high specific surface area and excellent electrical conductivity of CB facilitated rapid electron transfer, thereby significantly amplifying the signal intensity [25]. In addition, the unique electronic structure and nitrogen-rich framework of g-C_3_N_4_ provided abundant active sites, improving robust π-π stacking and hydrogen bonding interactions with CAR molecules [26]. Hence, this nanocomposite effectively minimized charge transfer resistance and promoted a highly stable and homogeneous distribution of the MIP layer, thereby optimizing the accessibility of target CAR analytes to the active recognition cavities. Finally, it enabled a lower LOD and enhanced analytical performance compared to conventional methods. The electrochemical sensor based on the CB-g-C_3_N_4_ nanocomposite has also garnered significant interest due to its minimal waste generation, establishing this novel strategy as a sustainable sensing platform and a significant advancement in the field of electrochemical sensors. This methodology was distinguished from alternative techniques by its rapid analysis speed and minimal sample volume requirements. Its practical utility was further evidenced by its capacity to detect pesticide residues in orange juice samples, which was essential for assessing health risks associated with metabolic disorders. In contrast, conventional chromatographic techniques such as HPLC [4] and GC-MS [5] typically required more complex sample preparation and longer analysis times for CAR determination for rapid on-site analysis. Moreover, this electrochemical sensor offered significant advantages over conventional commercial assay kits and rapid test strips, primarily in terms of superior analytical performance and robustness. Unlike commercial ELISA kits or lateral flow immunoassays, which often relied on costly, temperature-sensitive biological receptors (antibodies/enzymes) that were prone to denaturation, this MIP-based sensor provided exceptional chemical and thermal stability, lower production costs, and a longer shelf life. Despite these advantages, transitioning this technology from the laboratory to real-world applications involved significant challenges. These included the requirement for portable electrochemical workstations, the necessity for sample pretreatment to eliminate matrix effects in complex environmental or agricultural samples, and the need to improve the long-term reproducibility of electrode modifications under fluctuating field conditions.

### 3.6. Recovery

To assess the performance of the MIP/CB-g-C_3_N_4_/GCE electrode, recovery experiments were conducted on orange juice samples, yielding results close to 100.00%, as detailed in Table 2. These findings revealed the sensor’s high precision and superior selectivity in the presence of complex matrix components. Further validation was achieved by comparing these results with the standard gas chromatography–mass spectrometry (GC-MS) method [5]. The agreement was evaluated between the MIP/CB-g-C_3_N_4_/GCE electrode and the established GC-MS method (Table 2) by using the Kruskal–Wallis test. No statistically significant differences were observed between the two analytical approaches, confirming the reliability of the MIP/CB-g-C_3_N_4_/GCE electrode (KW_tabulated_ > KW_calculated_, *p* > 0.05). Moreover, the application of the standard addition method for CAR quantification in orange juice yielded reliable calibration data, expressed by the linear equation: y (μA) = 0.3011 × (C_CAR_, nM) − 0.183. The remarkable harmony between the slopes of the direct calibration and standard addition curves provided strong evidence of the high specificity of the MIP/CB-g-C_3_N_4_/GCE electrode, confirming the sensor’s robust precision and reliability.

### 3.7. Selectivity, Stability, and Reproducibility of MIP/CB-g-C_3_N_4_/GCE electrode

**Figure 6:**
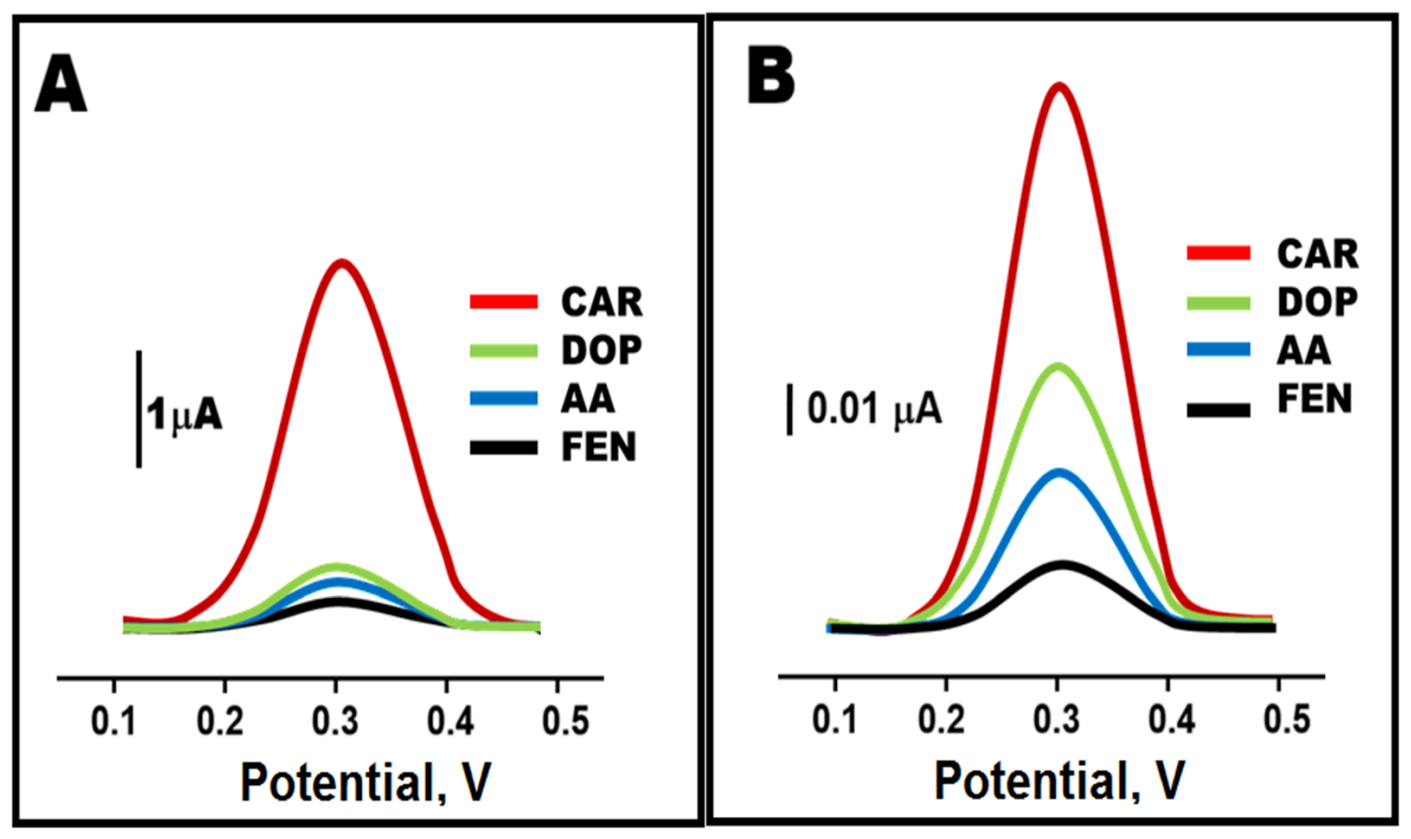
Square wave voltammograms recorded for two different electrodes: (**A**) the MIP/CB-g-C_3_N_4_/GCE electrode and (**B**) the NIP/CB-g-C_3_N_4_/GCE electrode, each measured separately in 10.0 nM CAR, 10.0 nM DOP, 10.0 nM AA, and 10.0 nM FEN.

Square wave voltammetry profiles for the MIP/CB-g-C_3_N_4_/GCE and NIP/CB-g-C_3_N_4_/GCE electrodes were illustrated in Figure 6, demonstrating their performances in the presence of 10.0 nM CAR and equimolar concentrations of interfering species including DOP, AA, and FEN. These compounds were selected as competing agents to evaluate the selectivity of the MIP/CB-g-C_3_N_4_/GCE toward CAR. DOP, AA, and FEN were chosen because of their high probability of co-occurrence with the CAR analyte in the same sample matrices (biological fluids, food, and environmental samples) as well as their strong electrochemical and/or structural interference potentials [24]. The peak currents recorded for each electrode (MIP and NIP) during individual exposure to CAR and the potential interfering species were analyzed. Subsequently, the superior specificity of the molecularly imprinted sensor was verified by the calculated selectivity (k) and relative selectivity (k′) coefficients shown in Table S3. These results indicated that the MIP-based electrode possessed a significantly enhanced affinity for CAR, exhibiting selectivity factors 10.0, 15.0, and 30.0 times higher than those for DOP, AA, and FEN, respectively. Furthermore, k′ values ranging from 3.00 to 5.00 confirmed the effectiveness of the imprinting approach in ensuring precise analytical detection. Generally, a k value exceeding 1.0 indicated a favorable affinity toward the target analyte, while a k′ value notably greater than 1.0 demonstrated that the enhanced selectivity was attributed to the molecular imprinting process rather than non-specific adsorption. Consequently, these elevated k and k′ values indicated the sensor’s robust molecular recognition capabilities. Finally, the imprinting factor (IF) was calculated (Table S3), and this was the ratio of the binding capacity of the MIP to that of the NIP (IF = ∆i _MIP_/∆i _NIP_). An IF of 30.00 for the target analyte (CAR) indicated that the imprinting process successfully created high-affinity cavities that were structurally and chemically complementary to the target. In addition, since the IF for CAR (30.00) was higher than the IFs for the structural analogs (DOP: 6.00, AA: 6.67, FEN: 10.00), the sensor showed a strong binding mechanism, which was critical for accurate diagnostic applications in complex biological matrices.

The long-term stability of one MIP/CB-g-C_3_N_4_/GCE electrode was systematically evaluated over a seven-week period by monitoring peak current responses to a 10.0 nM CAR solution. As illustrated in Figure S4, the MIP/CB-g-C_3_N_4_/GCE electrode exhibited exceptional stability, retaining approximately 99.04% of its initial peak current signal by the end of the seventh week.

**Figure 7:**
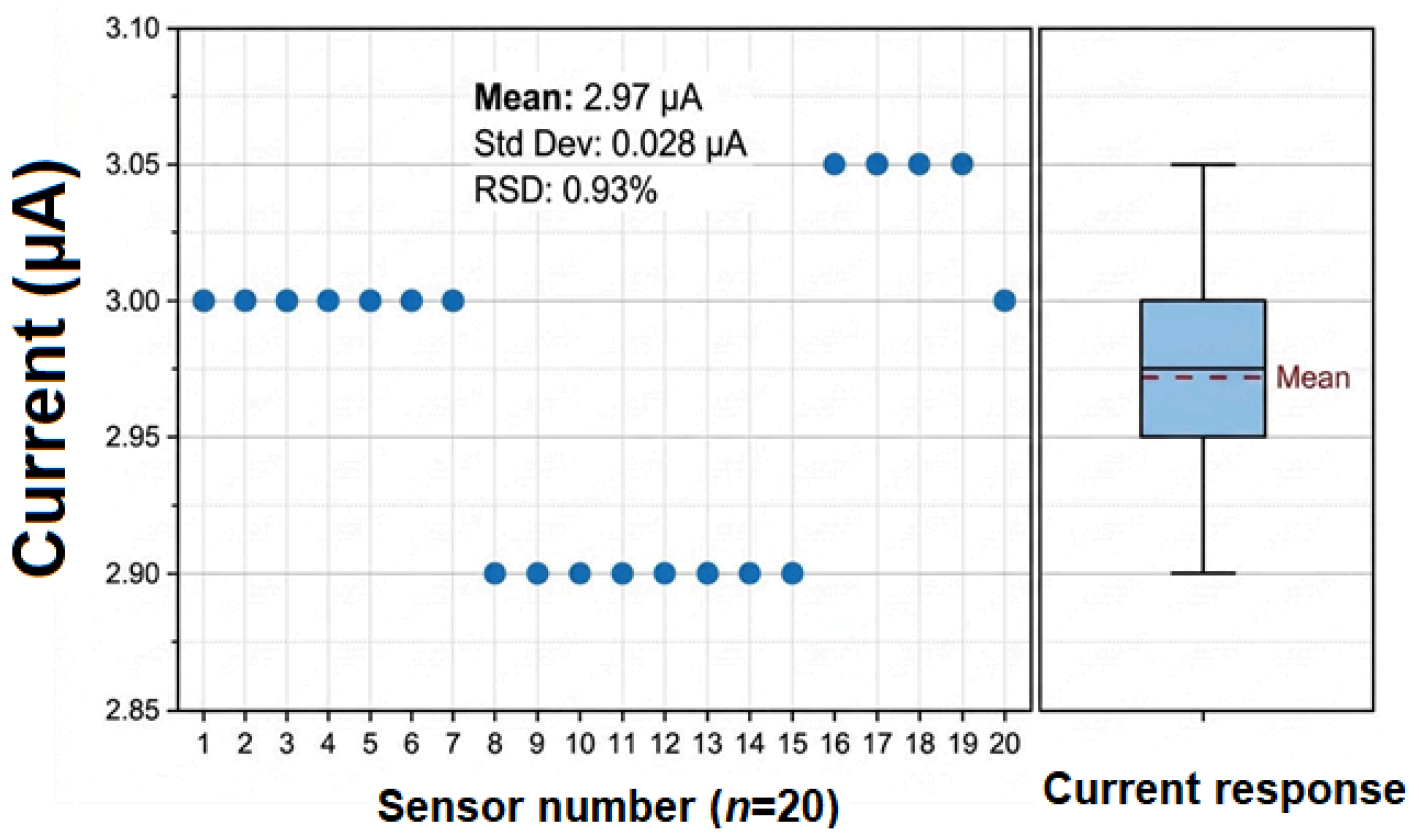
Individual response distribution of 20 independently prepared sensors for CAR detection.

To assess the fabrication consistency of the MIP/CB-g-C_3_N_4_/GCE electrode, the reproducibility was evaluated by recording the peak current responses of 20 independently prepared sensors in a 10.0 nM CAR solution. The reported relative standard deviation (RSD) value of 0.93% indicated high precision, with individual responses exhibiting a narrow and normal distribution centered around the mean current response. Figure 7 illustrates that the measured values consistently fall within a tight range, confirming the excellent reproducibility of the electrode manufacturing process.

### 3.8. Ruggedness and Robustness of MIP/CB-g-C_3_N_4_/GCE Electrode

To assess the reliability of the fabricated MIP/CB-g-C_3_N_4_/GCE, ruggedness and robustness evaluations were conducted, and the data were analyzed using the Wilcoxon test. The electrode’s ruggedness was confirmed by its consistent performance across six replicate analyses performed by two different analysts for the detection of 10.0 nM CAR; the lack of statistically significant differences (T_calculated_ > T_tabulated_, *p* > 0.05) confirmed its dependability. Additionally, the sensor demonstrated exceptional robustness by maintaining stable analytical performance despite slight fluctuations in experimental conditions—specifically pH variations between 6.9 and 7.1—and no significant deviations (T_calculated_ > T_tabulated_, *p* > 0.05) were observed.

### 3.9. Mathematical Analysis of MIPs Sensor and Thermodynamics

To evaluate the adsorption behavior, a systematic kinetic analysis using the pseudo-first-order model was performed on the MIP/CB-g-C_3_N_4_/GCE. To elucidate the equilibrium adsorption characteristics, three isotherm models such as Langmuir, Freundlich, and Langmuir–Freundlich were applied. The mathematical expressions of these models are provided below.

(2) Langmuir: ∆I = {∆I_max_ [A]/K_D_ + [A]}

(3) Freundlich: ∆I = ∆I_max_ [A]^1/n^

(4) Langmuir–Freundlich: ∆I = {∆I_max_ [A]^1/n^/K_D_ + [A]^1/n^}

The Langmuir, Freundlich, and Langmuir–Freundlich models were applied to experimental data. According to Table 3, the results exhibited high correlation coefficients (R^2^ > 0.99) for both the Langmuir and Langmuir–Freundlich models. The fact that the adsorption capacity reached saturation at a concentration of 50.0 nM and the calculated dissociation constants (K_D_) in the range of 40.00–45.45 nM confirmed that the developed polymer surface contained specific binding cavities with high affinity for CAR molecules. The heterogeneity factor (n or 1/n) values derived from the Langmuir–Freundlich model revealed that the energetic distribution of the binding sites on the MIP surface was predominantly consistent with a monolayer adsorption mechanism, but exhibited slight heterogeneity. Hence, these findings supported the potential of the developed sensor to exhibit high selectivity and analytical performance in CAR determination.

Since electrode preparation and analytical applications were conducted at 25 °C, the standard Gibbs free energy (ΔG°) at 25 °C was calculated using the coupling constant (K_A_) derived from the Langmuir isotherm, relating to the equation ΔG° = −RT ln(K_A_). The calculated ΔG° value for CAR interaction was found to be approximately −42.1 kJ/mol. This high negative value demonstrated that the binding process of CAR molecules to polymer cavities was spontaneous and thermodynamically highly favorable at 25 °C. The magnitude of the obtained ΔG° value (>−20 kJ/mol) indicated that the binding was not limited to simple physical adsorption; rather, it involved strong interactions—likely of a chemical nature, such as hydrogen bonding—between the specific recognition sites on the polymer surface and the CAR molecules.

### 3.10. Greenness Evaluation

The greenness profile of the MIP/CB-g-C_3_N_4_/GCE electrode was assessed using both the Green Analytical Procedure Index (GAPI) and the Analytical GREEnness metric (AGREE). The GAPI pictogram, which employed green, yellow, and red color segments, reflected the performance of all steps involved in the MIP/CB-g-C_3_N_4_/GCE electrode-based procedure. In parallel, the AGREE score, expressed on a scale from 0.0 to 1.0, was calculated as 0.91, indicating that the method was highly harmonious with green analytical chemistry principles (Figure S5), while the BAGI evaluation (Figure S6) further confirmed the high practical applicability of the MIP/CB-g-C_3_N_4_/GCE electrode.

## 4. Conclusions

In summary, this study successfully developed an electrochemical sensor for the precise quantification of CAR, utilizing a CB-g-C_3_N_4_ nanocomposite synthesized through an environmentally sustainable method. The developed sensor exhibited a linear dynamic range from 1.0 × 10^−9^ M to 5.0 × 10^−8^ M with a high detection limit of 3.0 × 10^−10^ M. Furthermore, the practical utility of the sensor platform was confirmed through recovery assays in orange juice samples, yielding results consistently close to 100.0%. Moreover, the accuracy of the developed sensor was verified through comparative analysis with the standard GC-MS method, revealing no statistically significant differences between the two techniques. Ultimately, the sensor’s good selectivity, long-term stability, and fabrication reproducibility established it as a robust analytical tool for the precise detection of CAR. To prove the more general applicability of the electrochemical sensor developed in this study, it can be applied to more real samples and used for routine food control analyses.

## Figures and Tables

**Table 1:** Comparative analytical performance of the MIP/CB-g-C_3_N_4_/GCE sensor and previously reported electrochemical methods for CAR determination.

Method/Material	Linear Range (M)	LOD (M)	Ref.
Co_9_S_8_@NSC/GCE	1.0 × 10^−5^–2.6 × 10^−4^	3.2 × 10^−9^	[24]
MIP/RGO@Au/GCE	5.0 × 10^−9^–4.0 × 10^−7^	2.0 × 10^−8^	[27]
PEDOT/PSS/GO/GCE	1.0 × 10^−6^–9.0 × 10^−5^	1.0 × 10^−7^	[28]
NdOCl/Nd_2_O_3_	3.0 × 10^−6^–2.0 × 10^−4^	8.1 × 10^−7^	[29]
Adsorptive stripping voltammetry	1.0 × 10^−7^–1.0 × 10^−5^	6.0 × 10^−8^	[30]
Carbon Nitride Dots and Poly(allylamine hydrochloride)	2.0 × 10^−7^–2.0 × 10^−6^	7.1 × 10^−6^	[31]
MIP/CB-g-C_3_N_4_/GCE	1.0 × 10^−9^–5.0 × 10^−8^	3.0 × 10^−10^	This study

**Table 2:** Recovery results of CAR (*n* = 6).

MIP/CB-g-C_3_N_4_/GCE				GC-MS	
Sample	Added CAR (nM)	Found CAR (nM)	* Recovery (%)	Found CAR (nM)	* Recovery (%)
Orange juice	-	3.87 ± 0.01	-	3.86 ± 0.02	-
	1.00	4.88 ± 0.02	100.21 ± 0.08	4.87 ± 0.02	100.21 ± 0.02
	10.00	13.86 ± 0.01	99.93 ± 0.06	13.85 ± 0.03	99.93 ± 0.02
	20.00	23.88 ± 0.04	100.04 ± 0.03	23.87 ± 0.01	100.04 ± 0.04
	30.00	33.88 ± 0.01	100.03 ± 0.02	33.87 ± 0.02	100.03 ± 0.01
	40.00	43.86 ± 0.01	99.98 ± 0.03	43.85 ± 0.01	99.98 ± 0.01

* Recovery = Found CAR, nM/Real CAR, nM.

**Table 3:** Langmuir, Freundlich, and Langmuir–Freundlich parameters.

Langmuir		Freundlich		Langmuir–Freundlich	
∆I_maks_	16.80 µA	∆I_maks_	-	∆I_maks_	17.10 µA
K_A_	0.025 nM^−1^	1/n	0.82	K_A_	0.022 nM^−1^
K_D_	40.00 nM	R^2^	0.9750	K_D_	45.45 nM
R^2^	0.9980			1/n	0.88
				R^2^	0.9990

## Data Availability

The original contributions presented in this study are included in the article/Supplementary Materials. Further inquiries can be directed to the corresponding author.

## Supplementary Materials

The following supporting information can be downloaded at https://www.mdpi.com/article/10.3390/foods15183313/s1, Figure S1. SEM images of (A) CB and (B) CB-g-C3N4 nanocomposite; Figure S2. SEM images of (A) MIP/CB-g-C_3_N_4_/GCE surface and (B) NIP/CB-g-C_3_N_4_/GCE surface; Figure S3. Effect of (A) pH, (B) mole ratio, (C) scan cycle, and (D) elution time on signals of square wave voltammograms in the presence of 10.0 nM CAR in 0.1 M, pH 7.0 phosphate buffer (*n* = 6); Figure S4. Stability test of MIP/CB-g-C_3_N_4_/GCE electrode in the presence of 10.0 nM CAR in 0.1 M, pH 7.0 phosphate buffer (*n* = 6); Figure S5. Method greenness assessment tools pictograms; Figure S6. Practicality assessment of MIP/CB-g-C_3_N_4_/GCE electrode using BAGI tools; Table S1. EDX results of the synthesized samples; Table S2. Data of the calibration curve for the proposed sensor; Table S3. k, k′ and IF values of MIP/CB-g-C_3_N_4_/GCE and NIP/CB-g-C_3_N_4_/GCE (*n* = 6).
