# Supplementary material for: Development of Carbofuran Imprinted Electrochemical Sensor Based on Carbon Black–Graphitic Carbon Nitride Nanocomposite and Carbofuran Detection in Orange Juice Samples
Source: Foods. 2026 Sep 19;15(18):3313. doi: 10.3390/foods15183313 (PMC13606741; doi:10.3390/foods15183313)
Supplement: Supplementary file 1 [file foods-15-03313-s001.zip › foods-4561746-supplementary.pdf]

Supplementary Data

For

Development of Carbofuran Imprinted Electrochemical Sensor Based on Carbon Black–Graphitic Carbon Nitride Nanocomposite and Carbofuran Detection in Orange Juice Samples

Didem Gün [1], Dilek Doğan [2], İlknur Polat [2], İsmail Hakkı Akbudak [3], Mehmet Lütfi Yola [4,5,*]

[1] Department of Basic Sciences, Faculty of Engineering, Hasan Kalyoncu University, Gaziantep 27010,
Türkiye; didem.gun@hku.edu.tr

[2] Department of Nutrition and Dietetics, Faculty of Health Sciences, Hasan Kalyoncu University, Gaziantep 27010, Türkiye; dilek.dogan@std.hku.edu.tr (D.D.); ilknur.polat@hku.edu.tr (İ.P.)

[3] Division of Intensive Care Medicine, Department of Internal Medicine, Faculty of Medicine, Pamukkale University, Denizli 20160, Türkiye; iakbudak@pau.edu.tr

[4] Department of Biology, Faculty of Science, Ankara University, Ankara 06100, Türkiye

[5] Integrated Technologies Research Center (BUTAM), Ankara University, Ankara 06690, Türkiye

* Correspondence: mehmetlutfiyola@ankara.edu.tr; Tel.: +90-3122168600; Fax: +90-3122868900

$$LOQ = 10.0\ S / m$$

$$LOD = 3.3\ S / m$$

S: Standard deviation of the intercept and m: Slope of the regression line

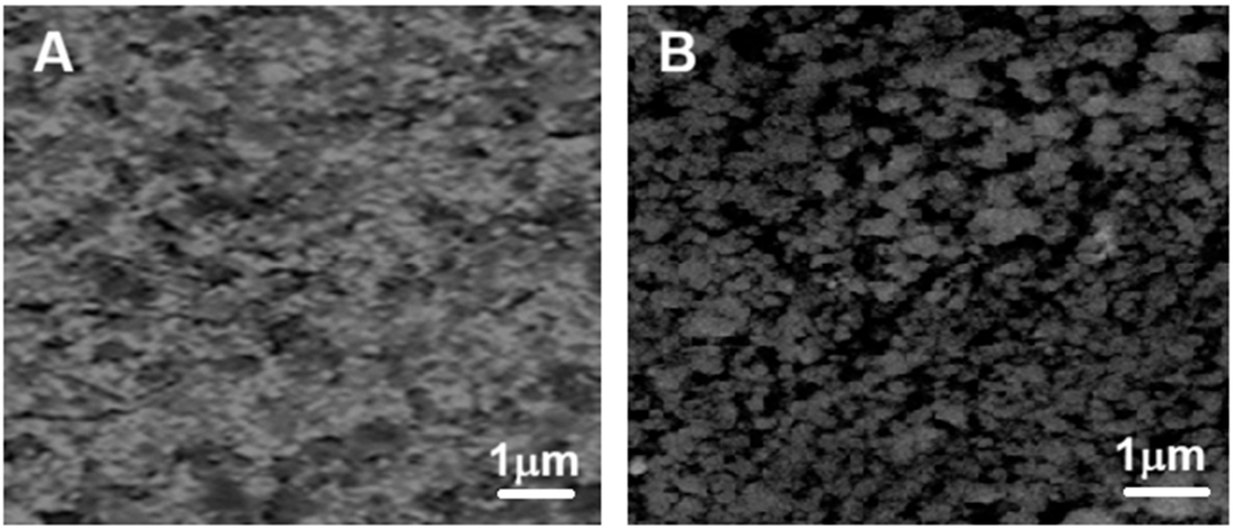

Figure S1. SEM images of (A) CB and (B) CB-g-C_3N_4 nanocomposite

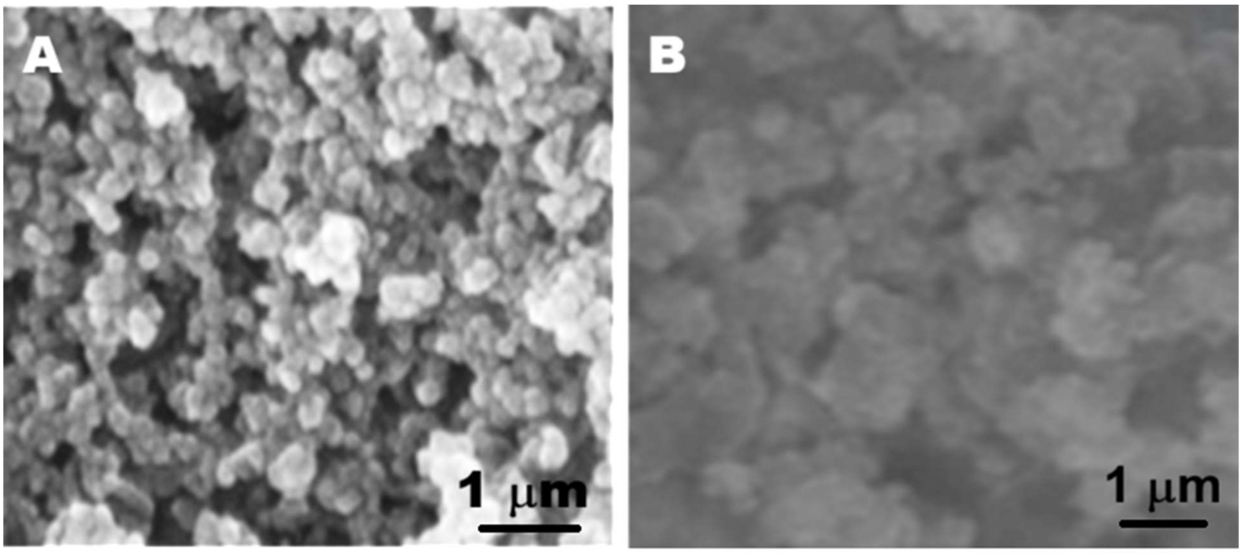

Figure S2. SEM images (A) MIP/CB-g-C_3N_4/GCE surface and (B) NIP/CB-g-C_3N_4/GCE surface

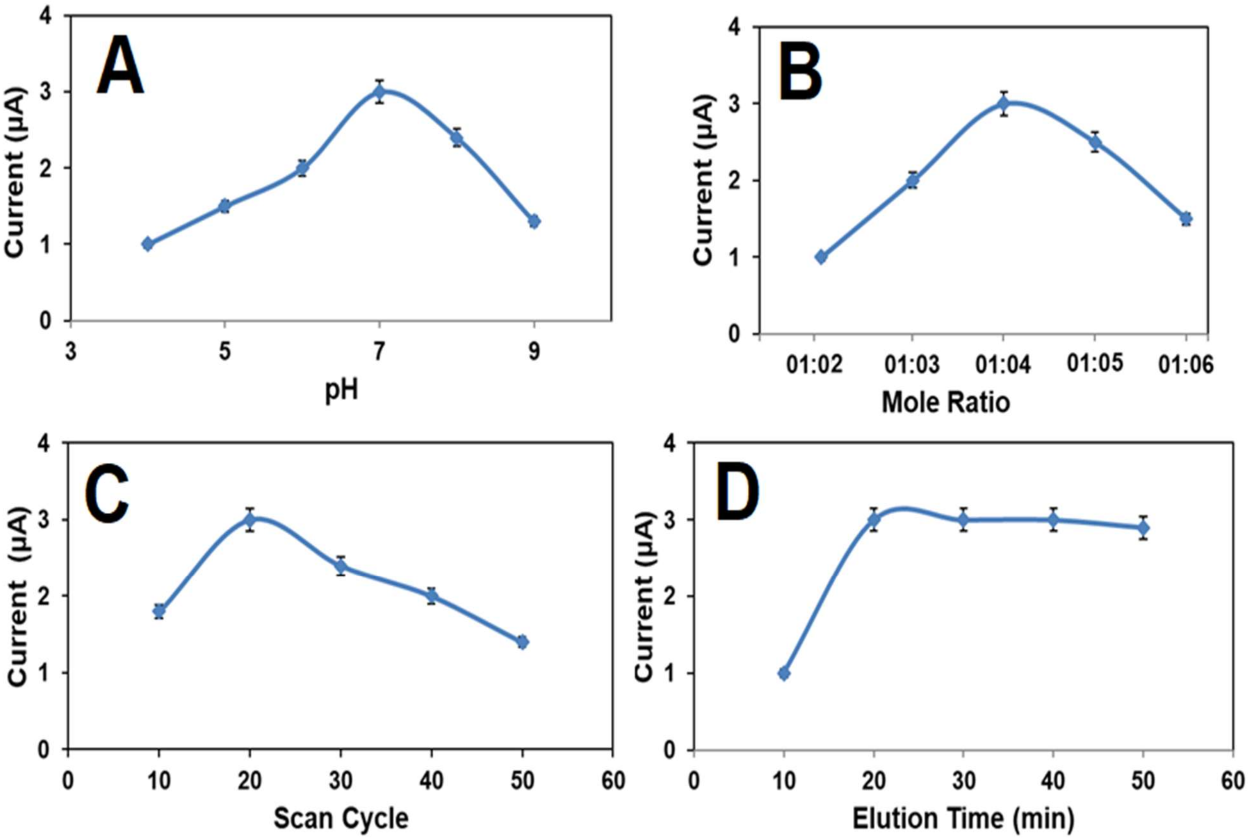

Figure S3. Effect of (A) pH, (B) mole ratio, (C) scan cycle and (D) elution time on signals of square wave voltammograms in the presence of 10.0 nM CAR in 0.1 M, pH 7.0 phosphate buffer (*n*=6)

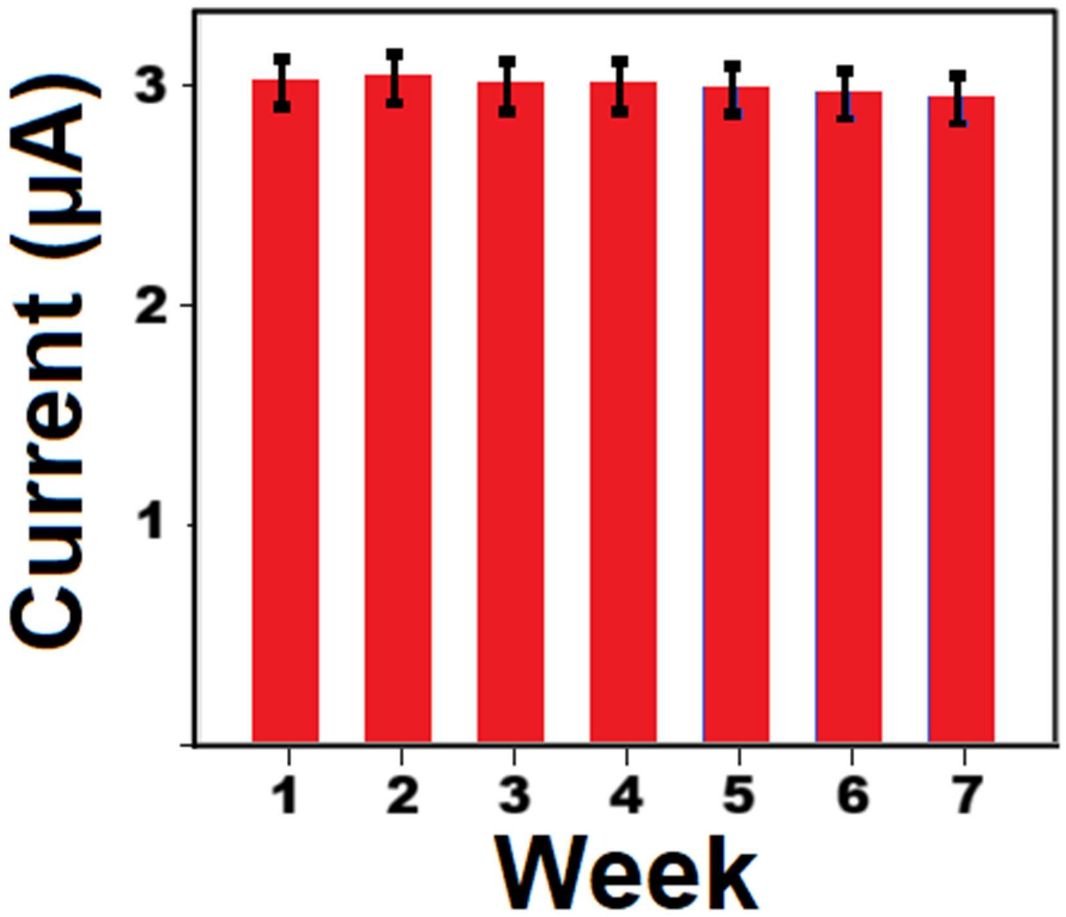

Figure S4. Stability test of MIP/CB-g-C_3N_4/GCE electrode in the presence of 10.0 nM CAR in 0.1 M, pH 7.0 phosphate buffer (*n*=6)

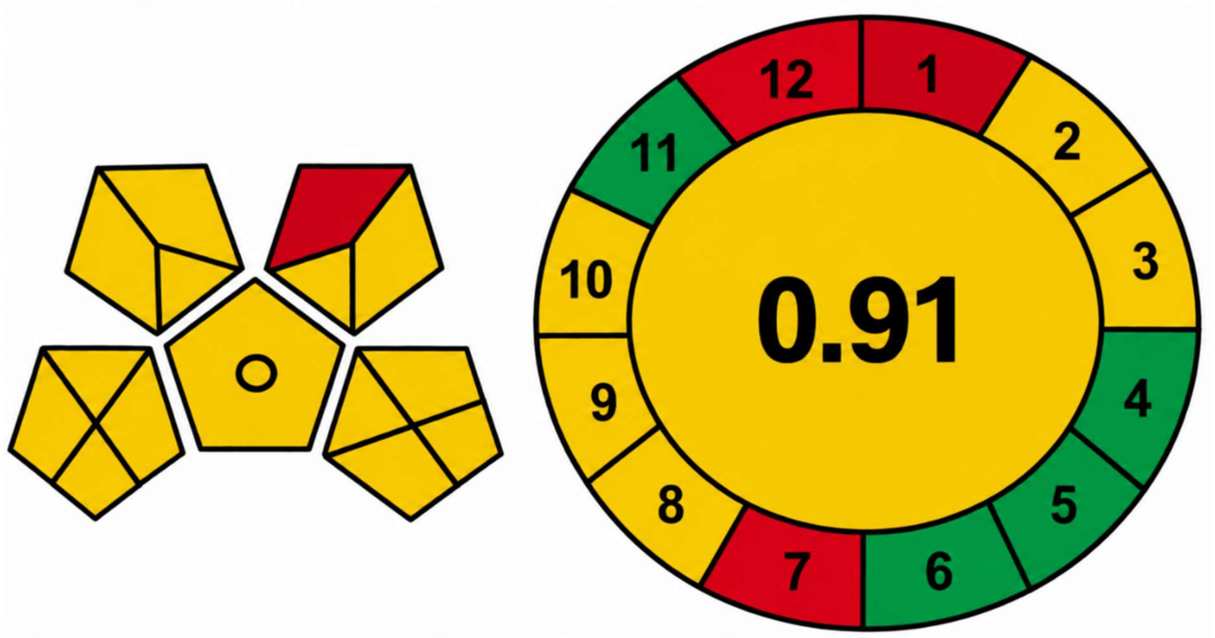

Figure S5. Method greenness assessment tools pictograms

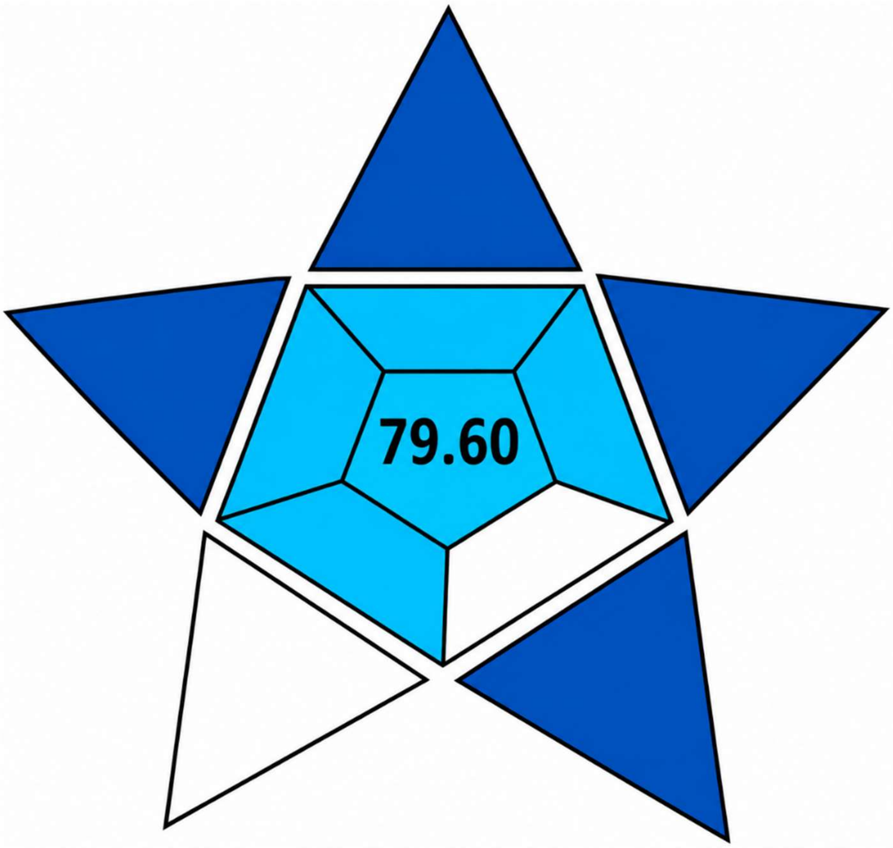

Figure S6. Practicality assessment of MIP/CB-g-C_3N_4/GCE electrode using BAGI tools

Table S1. EDX results of the synthesized samples

	Carbon	Nitrogen
**Samples**	**wt%**	**wt%**
g-C_3N_4	45.96	54.04
CB-g-C_3N_4	81.39	18.61
CB	96.25	3.75

Table S2. Data of the calibration curve for the proposed sensor

	*MIP/CB-g-C_3N_4/GCE
Regression equation	y (μA) = 0.3003x(C_{CAR}, nM) - 0.0287
Standard error of slope	0.22
Standard error of intercept	0.41
Correlation coefficient (r)	0.9998
Linearity range (M)	$1.0\times10^{-9} – 5.0\times10^{-8}$
Number of data points	6
LOQ (M)	1.0×10^{-9}
LOD (M)	3.0×10^{-10}

* The results were obtained using repeated measurements (*n*=6) with the same electrode

Table S3. k, k′ and IF values of MIP/CB-g-C_3N_4/GCE and NIP/CB-g-C_3N_4/GCE (*n*=6)

	**MIP**		**NIP**			
	**Δi**	**k**	**Δi**	**k**	**k′**	**IF**
CAR	3.000 ± 0.0001	-	0.100 ± 0.0002	-	-	30.00
DOP	0.300 ± 0.0002	10.00	0.050 ± 0.0001	2.00	5.00	6.00
AA	0.200 ± 0.0002	15.00	0.030 ± 0.0002	3.33	4.50	6.67
FEN	0.100 ± 0.0002	30.00	0.010 ± 0.0003	10.00	3.00	10.00

Analyte concentrations: 10.0 nM CAR, 10.0 nM DOP, 10.0 nM AA, and 10.0 nM FEN; IF = $\Delta\mathbf{i}_{MIP} / \Delta\mathbf{i}_{NIP}$
